# A Case of Wiedemann-Rautenstrauch Syndrome With Fatal Hyperkalemic Renal Failure

**DOI:** 10.7759/cureus.29320

**Published:** 2022-09-19

**Authors:** Mohamed A Ghamry, Rehab Salah, Eslam I Galal, Shereen Henin, Monica Dobs

**Affiliations:** 1 Pediatrics, Al-Salam Hospital, Ministry of Health, Port-Said, EGY; 2 Pediatrics, Benha University, Benha, EGY; 3 Internal Medicine/Pediatrics, California Institute of Behavioral Neurosciences & Psychology, Fairfield, USA; 4 Pediatrics, University of Florida College of Medicine – Jacksonville, Jacksonville, USA; 5 Medicine, Assiut University, Assiut, EGY

**Keywords:** neonatal death, neonatal kidney disease, senile apperance, wrs, polr3a, premature aging, rare genetic diseases, rapidly progressive renal failure, weidemann, porgeria

## Abstract

Wiedemann-Rautenstrauch Syndrome (WRS), also known as neonatal progeroid syndrome, is an extremely rare genetic syndrome characterized by a senile appearance at birth with multiple complex symptoms. We reported a case of a three-days old male neonate with features of WRS presented with fatal hyperkalemic renal failure which is a unique presentation not reported before in the cases affected with this syndrome. There is a positive family history of a previous sibling with the same features who suddenly died during the first week of life. This case report aimed to increase the awareness of WRS about the features and the importance of close follow-up of the affected cases, especially in the neonatal period among neonatal physicians.

## Introduction

Progeroid syndromes are a group of very rare genetic disorders characterized by clinical features of aging at an early age [1]. The Wiedemann-Rautenstrauch Syndrome (WRS) is a type of progeroid affection characterized by hypotrophy of subcutaneous fat and possibly other mesenchymal tissues. Several features of aging are evident at birth, hence it is referred to as a neonatal progeroid condition. WRS is a rare condition with fewer than 40 patients reported from the first case reported in 1977 until 2016 [2]. After 2016, five reports only could be found in the literature.

## Case presentation

A three-day-old term male neonate was the second child of healthy, young parents (mother 23 and father 27 years old) with positive consanguinity. Pregnancy was normal and non-complicated home vaginal delivery was reported. He presented to the neonatal emergency room with a complaint of decreased urination and poor feeding since birth. Physical examination showed an active crying baby with loss of subcutaneous fat, wide anterior fontanelle, sparse hair, and prominent scalp veins. The face appeared progeroid and triangular with a beak-shaped nose, thin erythematous dry skin, bilateral entropion, hypoplastic mandibular rami, and a high palate (Video 1). The neck was short with redundant skin folds while the chest examinations showed no apparent abnormalities and no cardiac murmurs. Abdominopelvic examination revealed cryptorchidism and no visceromegaly. His older brother had the same facial features and suddenly died in the first week of his life.

**Video 1:** Dysmorphic features of the reported Wiedemann Rautenstrauch Syndrome case showing senile characteristic face with deformed mandible and short neck

His documented weight in the emergency room was 2.7 Kg. During the initial assessment, the patient had a heart rate of 67/min, blood pressure of 50/35  mmHg, oxygen saturation of 95%, Respiratory rate of 47/minute, and temperature of 37°C. He was given epinephrine and placed on a dopamine drip. He rapidly developed difficulty in breathing and lethargy, then he was intubated. A urine catheter had been inserted but no urine passed. His hemoglobin 16.1 gm, leukocytes count 12 k/μL, neutrophils 58%, bands 7%, lymphocytes 39%, and platelets 394k/μL, serum potassium of 9.5  mEq/dl, sodium 120  mEq/l, bicarbonate 10 mEq/dl, blood urea nitrogen (BUN) 150mg/dl, and creatinine 8.7  mg/dl were reported and electrocardiogram (ECG) revealed bradycardia with elevated T wave and a wide QRS complex. A blood culture was drawn which later showed no growth. The baby was initially treated with antibiotics, sodium chloride, sodium bicarbonate, calcium, insulin, and dextrose along with dialysis kit preparation. He simultaneously developed asystole and trials of cardiopulmonary resuscitation failed. The parents refused ultrasound, autopsy, or genetic analysis. 

## Discussion

Wiedemann-Rautenstrauch syndrome (WRS) was first reported in 1977 by Rautenstrauch who reported on two sisters with progeria-like syndrome [3]. In 1979 Wiedemann described two unrelated males with the same condition [3]. Hence the name of Wiedemann-Rautenstrauch syndrome.

Autosomal recessive mutations in the POLR3A gene located on the long arm of chromosome 10 (10q22.3) have been associated with WRS [2].

WRS represents a complex of variable symptoms and signs. However, the characteristics of premature aging or progeroid features since birth remain enough to allow a secure diagnosis [3]. Table 1 shows a comparison between the clinical features of our case and some of the previously reported cases.

**Table 1:** Comparison of the clinical features of some of the previously reported cases and our current reported case NR= Not reported, M= male, F= female, += positive, -= negative

	Wiedemann [4]		Rautenstrauch, et al. [5]		Devos, et al. [6]	Rudin, et al. [7]	Obregon, et al. [8]	Bitoun, et al. [9]	Arboleda, et al. [10]			Beauregard-Lacroix, et al. [11]	The current reported case
Birth weight in grams	2200	2550	2380	2110	2110	2500	2300	1950	1500	1700	2120	910	2700 on the 3^rd^ day
Birth length in centimeters	45	49	48	47	48	45	NR	42	43	45	47	32	51 on the 3^rd^ day
Sex	M	M	F	F	F	M	F	M	F	M	M	F	M
Consanguinity	-	-	-	-	+	-	-	+	-	-	-	-	+
Senile appearance	+	+	+	+	+	+	+	+	+	+	+	+	+
Psudohydrocephalus	+	+	+	+	+	+	+	+	+	+	+	-	-
Wide open sutures	+	+	+	+	+	+	+	+	+	+	+	NR	+
Widened fontanelles	+	+	+	+	+	+	NR	+	+	+	+	+	+
Sparse scalp hair	+	+	+	+	+	+	+	-	+	+	+	NR	+
Prominent scalp veins	+	+	+	+	+	+	+	+	+	+	+	NR	+
Hypoplasia of the facial bones	+	+	+	+	+	+	+	+	+	+	+	+	+
Low set ears	+	+	+	+	+	+	+	+	+	+	+	+	-
Beak shaped nose	+	+	+	+	+	+	+	-	+	+	+	NR	+
Dentition present at birth	2	4	2	2	1	4	2	-	1	4	4	NR	-
Large hands and feet with long fingers and toes	+	+	+	+	+	+	+	NR	+	+	+	+	-
Prepucial hypoplasia	NR	NR				NR		-		+	+		-
Large penis	+	+				NR		-		-	-		-
Cryptorchidism	NR	NR				+		-		+	+		+
Gynaecomastia	NR	NR				NR		-		+	+		-
Lipoatrophy	+	+	+	+	+	+	+	+	+	+	+	-	+
Buttocks' fat pads	+	+	NR	NR	+	+	+	-	+	-	NR	NR	-

The average survival in WRS is seven months, although survival into the third decade of life has been reported [12]. WRS patients have a short life expectancy due to malnutrition (leading to hypolipidemia and hypoalbuminemia) or after severe infection [13], but no previously reported cases of death due to hyperkalemia or renal failure. WRS cases suffer from failure to thrive despite high-caloric nutrition with no reported cause [13]. The prognosis of WRS may be dependent on the presence and severity of mental and/or neurological impairment [13]. The mental status in WRS patients ranges from normal to mild-to-moderate mental retardation but the latter is more common [13].

Our case has a dramatic rapid fatal progression and no clues to determine the exact cause of hyperkalemia oliguric or anuric renal failure, especially with the parents' refusal of further genetic and/or radiological investigations. Reporting more patients will be needed to define if this syndrome may be associated with structural renal abnormalities and to determine this syndrome's natural course and prognosis as well as increase knowledge of its pathophysiology.

## Conclusions

This report relates the case history of a neonate who demonstrated the typical symptoms of Wiedemann-Rautenstrauch Syndrome at birth and develops fatal anuric hyperkalemic renal failure on his third day of life. The aim of the report is to raise awareness among health care providers about the clinical features of this syndrome and the importance of assessing the vital organs' functions of the affected cases to prevent possible life-threatening conditions. More reports are needed to stand on the nature, pathogenesis, and prognosis of this rare syndrome.
